# Susceptibility of human neural stem cells to SARS-CoV-2: Entry mechanisms and glycocalyx influence

**DOI:** 10.1371/journal.pone.0360734

**Published:** 2026-10-21

**Authors:** Ann Song, Cori Zuvia, Prue Talbot

**Affiliations:** 1 Department of Molecular, Cell, and Systems Biology, University of California, Riverside, California, United States of America; 2 Department of Neuroscience, University of California, Riverside, California, United States of America; University of Texas Medical Branch at Galveston, UNITED STATES OF AMERICA

## Abstract

Previous work demonstrated that ectodermal cells exhibit greater susceptibility to SARS-CoV-2 than endodermal and mesodermal cells, raising concerns about potential vulnerability of the developing nervous system. We hypothesized that neural stem cells (NSCs) derived from human ectoderm are susceptible to SARS-CoV-2. Using pseudotyped viral particles representing both wild-type and Omicron spike variants, we confirmed efficient transduction in NSCs, with Omicron variants preferentially utilizing endocytosis-mediated entry. Inhibition of endocytosis with filipin and OcTMAB significantly reduced viral entry across all spike variants. Low glycosylation levels on NSCs facilitated viral entry, and enzymatic removal of glycosylation increased their susceptibility. Ectodermal infection by SARS-CoV-2 raises serious concern for potential teratogenic effects on the nervous system, possibly causing latent or subclinical anomalies not immediately evident at birth. Therefore, future clinical studies and long-term surveillance of infants and children exposed *in utero* are necessary to investigate and identify potential neurodevelopmental deficits.

## Introduction

Human embryos and fetuses are highly susceptible to infectious agents such as viruses, which are well established teratogens [1]. SARS-CoV-2 infection during early embryonic development is of particular concern, because infected embryos may fail to survive and those that do survive may have severe developmental anomalies. Our previous research showed that human embryonic stem cells (hESCs), which model the epiblast [2,3], as well as germ layer cells are susceptible to SARS-CoV-2 [4]. Susceptibility to SARS-CoV-2 varied among the different cell types with ectoderm being the most susceptible [4]. Such elevated susceptibility in ectodermal cells raises the possibility of subtle or delayed neurodevelopmental disturbances that may not be evident at birth.

In Song et al., 2025 [4], we performed trilineage differentiation of H9 hESCs into ectoderm, endoderm, and mesoderm. Quantitative flow cytometry analysis of transduction revealed that ectoderm achieved the highest infectability, while the endoderm and mesoderm showed substantially reduced transduction efficiency despite expressing comparable ACE2 levels. Glycosylation profiling using a lectin panel further showed that ectoderm had the sparsest glycocalyx (strong WGA binding indicating accessible sialic acids, but minimal binding to other glycans), while the mesoderm showed dense broad lectin binding, indicative of a heavily glycosylated surface. Endoderm displayed an intermediate lectin-binding pattern. These observations led to the hypothesis that glycocalyx density restricts viral access to ACE2 and hence reduces viral entry. To test this, we used neuraminidase to enzymatically remove sialic acids, the primary glycan determinant of WGA binding. Deglycosylation significantly increased SARS-CoV-2 entry across all germ layers, demonstrating a causal role for surface glycosylation in viral tropism. These embryonic-stage findings raise the question of whether ectoderm-derived cells, particularly neural progenitors, retain heightened susceptibility to SARS-CoV-2.

In adults, neurological effects of SARS-CoV-2 are well documented and involve both the central and peripheral nervous systems, which originate from the ectoderm [5]. Reported symptoms include anosmia and ageusia [6–8], Guillain-Barré syndrome [9,10], and encephalopathy [11,12]. Moreover, individuals with long COVID frequently experience persistent neurological symptoms such as cognitive impairment (“brain fog”), fatigue, and sleep disturbances [13,14]. Given our previous findings that ectoderm is the most susceptible germ layer to SARS-CoV-2 [4] paired with the established effects of SARS-CoV-2 on adult neural function, the derivatives of the ectoderm may be susceptible to SARS-CoV-2.

Evidence of vertical transmission further highlights the potential impact of SARS‑CoV‑2 on developing neural tissues and raises concerns about the vulnerability of ectodermal derivatives in embryos and fetuses. Vertical transmission of SARS-CoV-2 during the late third trimester is well documented, as demonstrated by viral detection in postpartum fetal samples [15,16]. Although some studies report that vertical transmission is rare [17,18], these conclusions may reflect suboptimal timing for qPCR detection [19] or the use of non-responsive endpoints. The strongest evidence of vertical transmission comes from a first-trimester stillbirth study [20], in which SARS-CoV-2 was detected in the placenta and multiple fetal organs, indicating direct fetal infection. Another first-trimester study involving SARS-CoV-2 positive pregnant women [21] reported that 30% of embryos/fetuses and 20% of syncytiotrophoblasts tested positive for viral RNA, clearly demonstrating viral transmission through the placental membrane. The lack of information on infection of ectodermal derivatives emphasizes the urgency of our research, which addresses this knowledge gap by utilizing a disease-in-a-dish model to demonstrate susceptibility of neural stem cells, an ectodermal derivative, to SARS-CoV-2 in earlier pregnancy stages.

The purpose of this study was to test the hypothesis that ectoderm-derived neural stem cells (NSCs) are more susceptible to SARS-CoV-2 than human embryonic cells. Specifically, we evaluated SARS-CoV-2 spike pseudoparticle entry by comparing the transduction efficiency of hESCs, ectoderm, and NSCs. Given Omicron’s global dominance and high transmissibility [22–24], we used Omicron (BA.1, BA.2) pseudoparticles alongside WT pseudoparticles, which we characterized previously [4,25]. SARS-CoV-2 can enter cells by two pathways: (1) TMPRSS2-mediated membrane fusion and/or (2) endocytosis. We assessed pathway usage with inhibitors specific to TMPRSS2 or clathrin-mediated and caveolae-dependent endocytoses. Finally, the effect of cell surface glycocalyx on viral entry was examined experimentally. Together, our findings provide mechanistic insight into SARS-CoV-2 entry into early neural progenitors and offer potential therapeutic strategies for mitigating the risk of vertical transmission.

## Materials and methods

### Tissue culture and reagents

H9 hESCs were obtained from WiCell (Madison, WI), which provided cell line validation information. The use of these cells and all experimental protocols were approved by the UCR Institutional Review Board (IRB protocol number 13106, review category exempt). All methods used with hESCs were carried out in accordance with relevant guidelines and regulations. Informed consent was obtained from the donor, whose identity is not known to us. Cells were grown in complete mTeSR Plus culture medium (STEMCELL Technologies, Vancouver, Canada) as described in our previous work [4,25,26]. HEK 293T cells and an ACE2-overexpressing cell line (HEK 293T-ACE2) (ATCC, Manassas, VA) were grown in DMEM with high glucose and 10% FBS (Gibco, Carlsbad, CA) [4,25,27].

The following small molecule inhibitors were used to target TMPRSS2: Ambroxol (10 µM; TCI Chemicals, Portland, OR), Aprotinin (10 µM; Tocris, San Diego, CA), Camostat (10 µM; Sigma-Aldrich, Burlington, MA), Nafamostat (10 µM; TCI Chemicals, Portland, OR). Endocytosis inhibitors that were used included: Dyngo4a (20 µM, Selleckchem, Houston, TX), Pitstop2 (20 µM; Abcam, Cambridge, MA), OcTMAB (10 µM; Abcam, Cambridge, MA), MiTMAB (10 µM; Abcam, Cambridge, MA), mβCD (20 µM; Sigma-Aldrich, Burlington, MA), Nystatin (20 µM; Sigma-Aldrich, Burlington, MA), and Filipin (20 µM; Sigma-Aldrich, Burlington, MA).

### Neural stem cell differentiation

In prior work, H9 hESCs were differentiated to ectoderm using the STEMdiff Trilineage kit ectoderm medium [4]. The current work extends these observations to more mature neural stem cells (NSCs) derived from the same differentiation lineage. To simulate early neural development, neural stem cell differentiation was performed with H9 hESCs using the STEMdiff^TM^ SMADi neural induction kit (STEMCELL Technologies, Vancouver, Canada) according to the manufacturer’s protocol. Single hESCs were first obtained using the Gentle Cell Dissociation Reagent (STEMCELL Technologies, Vancouver, Canada). 2 × 10^6^ cells were plated on Matrigel-coated 6-well plates in mTeSR Plus medium (STEMCELL Technologies, Vancouver, Canada) with ROCK inhibitor (10 µM; Tocris, San Diego, CA). Every 24 hours a medium change with neural induction medium was performed for 7 days. On the 7^th^ day, 2 × 10^6^ cells were dissociated using Accutase (Innovative Cell Technologies, San Diego, CA) then passaged onto a Matrigel-coated 6-well plate. Three passages were performed then cells were transferred to the STEMdiff^TM^ neural progenitor medium (STEMCELL Technologies, Vancouver, Canada) and cultured according to the manufacturer’s protocol.

### Fluorescence microscopy

The NSCs were seeded in 8-well chamber slides (Ibidi; Gräfelfing, Germany) and were fixed at room temperature in 4% paraformaldehyde in PBS for 15 min, then washed with PBS. For ACE2 and TMPRSS2 labeling, cells were treated with 50 mM DTT and 6 M guanidine-HCl for 5 minutes prior to quenching with 100 mM iodoacetamide. To verify that ACE2 and TMPRSS2 were located on the cell surface, fixed cells were labeled with the antibody and fluorescence lectin conjugate (WGA-FITC 1:100; Vector Laboratories, Newton, CA, USA).

For labeling with other antibodies, NSCs were permeabilized with 0.1% Triton X-100 for 10 min, then blocked with 10% donkey serum. Primary antibodies were diluted in blocking buffer. The following primary antibodies were used: mouse anti-PAX6 (1:60; Developmental Studies Hybridoma Bank, Iowa City, IA), mouse anti-NESTIN (1:200; Santa Cruz Biotechnology, Dallas, TX), mouse anti-OCT4 (1:200; Santa Cruz Biotechnology, Dallas, TX), goat anti-ACE2 (1:200; R&D Systems, Minneapolis, MN), and mouse anti-TMPRSS2 (1:200; Santa Cruz Biotechnology, Dallas, TX). Following overnight incubation with primary antibody at 4 °C, the cells were washed three times with 0.2% Tween in PBS, then incubated in the dark with Alexa Fluor-conjugated fluorescent secondary antibodies (1:500; Invitrogen, Carlsbad, CA). After washing in PBS, the cells were mounted with diluted Vectashield and DAPI for nuclear staining. A Nikon Eclipse Ti inverted microscope was used to image cells at 40x. NIS Elements Software and ImageJ software were used to collect images and analyze the data.

### TMPRSS2 activity assay

The TMPRSS2 cleavage assay was performed as described in our previous work [4]. A TMPRSS2 fluorogenic substrate, Boc-Gln-Ala-Arg-AMC HCl (2.5 mM, Bachem, Torrance, CA), was prepared in 50 mM Tris (pH 8) and 150 mM NaCl. After differentiation, NSCs were washed twice with PBS and lysed for 1 min on ice in RIPA buffer. The cells were then sheared with a 21-gauge needle, followed by centrifugation at 3,000 rpm for 5 min at 4 °C. The lysate protein was quantified using the Pierce BCA assay kit (Thermo Scientific, Waltham, MA). 10 µg of protein was added to each reaction well. Cells were preincubated with TMPRSS2 inhibitors for 24 hours before measuring enzymatic activity. The fluorogenic substrate was added to each well at a final concentration of 10 µM. Fluorescence intensity was measured at 340/440 nm using a BioTek Synergy HTX, multi-mode microplate reader (Winooski, VT).

### Lentiviral production to generate SARS-CoV-2 pseudoparticles

SARS-CoV-2 pseudoparticles were generated as described in our previous work [4,25,27]. HEK293T cells were plated with antibiotic-free medium at a density of 7 × 10^6^ cells in a T75 flask and transfected using lentiviral plasmids (Table 1) and a Lipofectamine3000 Kit (Invitrogen, Carlsbad, CA) according to the manufacturer’s protocol. After overnight incubation, fresh medium was added to cells supplemented with 1% BSA. Conditioned medium was collected and centrifuged 48 h post-transfection. The supernatant was filtered using a 0.45 µm Acrodisc syringe filter (Cytivia Life Sciences, Marlborough, MA), and the filtrate was mixed with 5x polyethylene glycol (Abcam, Cambridge, UK) and precipitated overnight at 4^o^C. The lentivirus was collected by centrifugation, and the pellet was resuspended in Viral Re-suspension Solution (Abcam, Cambridge, UK). Virus aliquots were stored at −80 °C. Prior to use in experiments, the transduction efficiency of each batch of viruses was tested in HEK 293T-ACE2.

**Table 1:** List of lentivectors and their amounts for a 75-cm^2^ flask.

Plasmid Name	Amount (µg)
pHAGE2-CMV-ZsGreen-W	7.5
HDM-Hgpm2	1.65
pRC-CMV Rev1b	1.65
HDM-tat1b	1.65
pcDNA3.3_CoV2_D18 (wild-type) or pcDNA3.3_SARS2_omicron_BA.1 (omicron BA.1 variant) or pcDNA3.3_SARS2_omicron_BA.2 (omicron BA.2 variant)	2.55

### Infection with human SARS-CoV-2 pseudoparticles

SARS-CoV-2 pseudoparticle infection was performed as described in our previous work [4,25,27]. 1.25 × 10^5^ of NSCs or H9 hESCs were seeded in 48-well plates, then infected 24 hours later with SARS-CoV-2 pseudoparticles using a multiplicity of infection (MOI) of 0.1. H9 hESCs served as an undifferentiated control. The medium was replaced after overnight incubation of the transduced cells. Cells were dissociated with Accutase 48 h post-transduction and washed three times with 0.5% BSA in PBS. After the final wash, the cells were resuspended in the same wash buffer and analyzed using flow cytometry.

Cells were analyzed using flow cytometry (Novocyte flow cytometer; Agilent Technologies, Santa Clara, CA) to quantify the percentage of cells expressing ZsGreen, an indicator of successful pseudoparticle transduction [4,25]. Flow cytometry gating was performed using NovoExpress software, and mock-infected cells served as background controls for each experiment. Data are presented as relative infection, or percentage of ZsGreen+ cells normalized to DMSO vehicle control (set to 100%). The resulting flow cytometer files were analyzed using NovoExpress software. Mock infection was used as a background control. In inhibitor experiments, cells were preincubated for 2 h with TMPRSS2 inhibitors or endocytosis inhibitors, which were maintained in the medium until harvesting for flow cytometry. Baseline infection assays and other experiments not designed to test inhibitor effects were performed in parallel without the addition of TMPRSS2 or endocytosis inhibitors.

### Endocytosis assay

NSC differentiation was performed in 8-well chamber slides (Ibidi; Gräfelfing, Germany). The cells were incubated overnight with TRITC-conjugated dextran (Invitrogen, Carlsbad, CA) with and without endocytosis inhibitors. After fixation in 4% paraformaldehyde, mounting was performed using diluted Vectashield with DAPI, and images were collected using a Nikon Eclipse inverted microscope.

### Cell surface glycosylation and deglycosylation assay

To determine surface glycosylation patterns, NSCs were labeled with various lectins (Con A, DBA, PNA, RCA120, SBA, UEA I, WGA from Vector Laboratories, Newton, CA, USA). After lectin labeling, cells were fixed in 4% paraformaldehyde for 15 minutes at room temperature and mounted in Vectashield containing DAPI for microscopy.

For deglycosylation, NSCs were seeded in 48-well plates. After 24 hours, the cells were treated with neuraminidase (Sigma-Aldrich, Burlington, MA) in DMEM at 37°C for variable periods of time (0 min, 45 min, 90 min, 180 min, 360 min). 1 Unit of neuraminidase liberates 1.0 µM of N-acetyl neuraminic acid per minute at 37°C using bovine submaxillary mucin. After deglycosylation, neuraminidase was quenched with culture medium, and cells were washed with PBS.

To assess SARS-CoV-2 entry, cells were transferred to neural maintenance medium, and SARS-CoV-2 pseudoparticles were added after the glycosidase treatment to allow transduction. After 48 hours, cells were collected and analyzed for viral entry using flow cytometry.

### Data analysis and statistics

For transduction assay and TMPRSS2 activity data, the mean and standard error of the mean for three independent experiments were plotted using GraphPad Prism 10 software (GraphPad, San Diego, CA). For transduction efficiency, the mean of the DMSO group was set to 100, and the inhibitor groups were compared to this value. Statistical significance was determined using Minitab Statistics Software (Minitab, State College, PA). When the data were not normally distributed, they were subjected to a logarithmic, arcsine square root, or Box-Cox transformation, and the data were retested to confirm that they satisfied the analysis of variance (ANOVA) model (normal distribution and homogeneity of variances). SARS-CoV-2 entry was analyzed by performing a one-way ANOVA, while TMPRSS2 cleavage analysis was performed using a two-way ANOVA, in which the factors were time and treatment. When the ANOVA means were significant (p < 0.05), groups treated with inhibitors were compared to the control group using Dunnett’s or Tukey post hoc analysis.

## Results

### NSC differentiation and characterization

NSC differentiation from H9 hESCs was successful as the pluripotency marker OCT4 was absent and the neural markers PAX6 and NESTIN were present (Fig 1A-C). WGA, a cell surface-binding lectin (Fig 1D and H), and ACE2 and TMPRSS2, the machinery for SARS-CoV-2 entry, were present and often aggregated into clusters on NSCs (Fig 1E and 1I). Surface localization of ACE2 and TMPRSS2 was confirmed by colocalization with WGA, which generated yellow fluorescence in clustered regions (Fig 1F and 1J). ACE2 and TMPRSS2 were localized in the plasma membrane both as clusters above the nuclei and as individual proteins (small puncta) away from the nuclei (Fig 1G and 1K). In addition, ACE2 and TMPRSS2 were often colocalized as indicated by the yellow fluorescence in many of the puncta in the merged images (Fig 1 L-N). These data confirm successful differentiation of H9 hESCs into NSCs that express the SARS-CoV-2 entry machinery ACE2 and TMPRSS2 at the cell surface.

**Fig 1:**
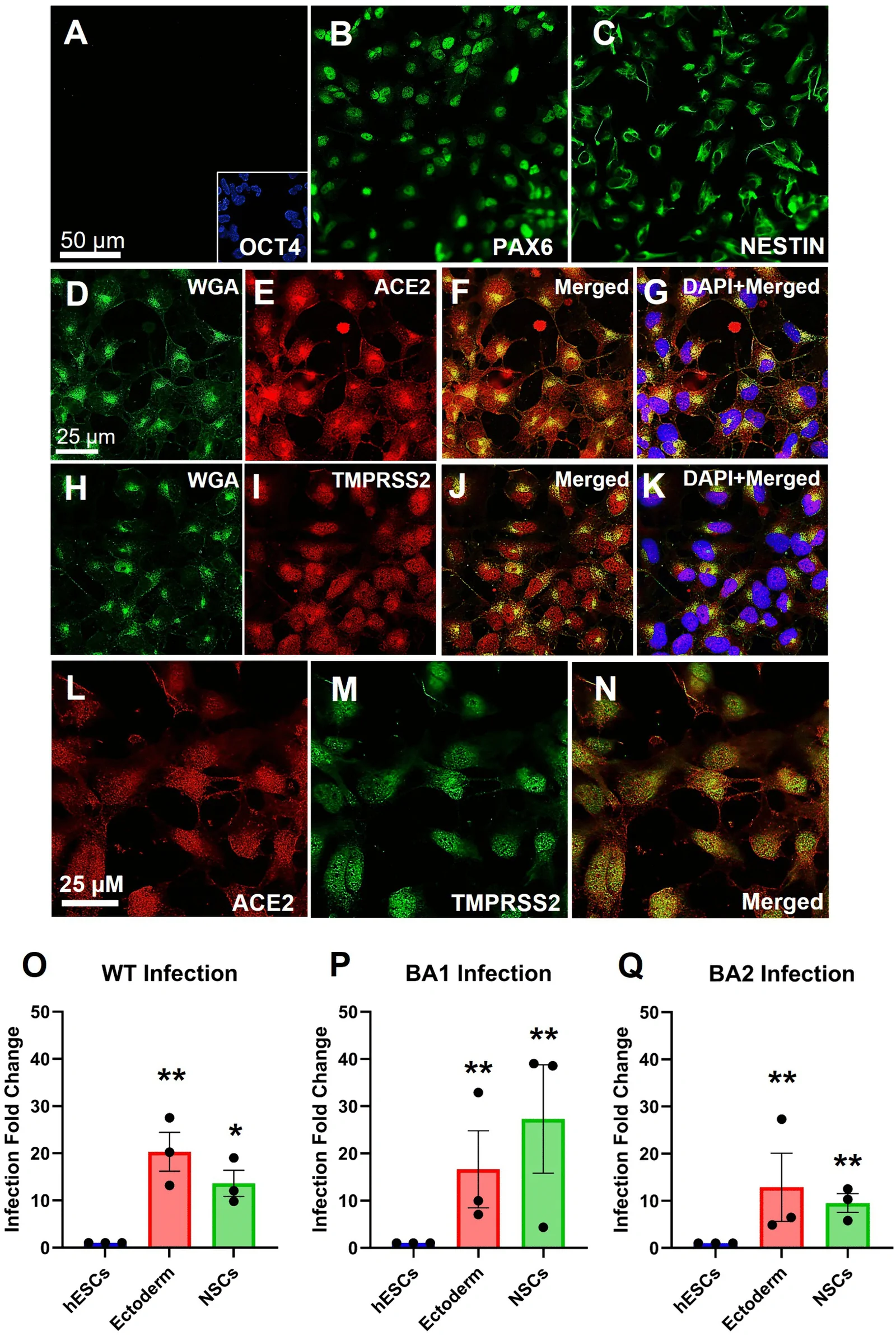
NSCs were susceptible to SARS-CoV-2 pseudoparticles. Immunocytochemistry showing successful differentiation of H9 hESCs into NSCs using (A) OCT4, (B) PAX6, and (C) NESTIN labeling. Immunocytochemistry showing (E) ACE2 and (I) TMPRSS2 localization in NSCs. Both samples were labeled with WGA conjugated to FITC to verify surface localization of (D) ACE2 or (H) TMPRSS2. Merged images of (F) WGA and ACE2 and (J) WGA and TMPRSS2 show colocalization. Colocalization of (L) ACE2 and (M) TMPRSS2 is shown in (N) the merged image. Representative images of three independent experiments are shown. Flow cytometry was performed to check the relative infection in NSCs with (O) WT spike pseudoparticles, (P) BA.1 pseudoparticles, and (Q) BA.2 pseudoparticles. Ectoderm and NSCs were not significantly different from each other in any group. One-way ANOVAs were performed on raw data, and the Tukey post-hoc test was used to compare to the hESCs control. Data are the means ± SEM of three independent experiments. * = p < 0.05, ** = p < 0.01.

### Variant-dependent pseudoparticle entry into hESCs, ectoderm, and NSCs

For each spike protein pseudoparticle (WT, BA.1, and BA.2), the ectoderm had significantly higher transduction efficiency (Relative infection) than the undifferentiated hESCs (Fig 1O-Q), in agreement with our prior research with WT pseudoparticles [4] and further showing that the BA.1 and BA.2 variants also entered the ectoderm. NSCs were also successfully transduced by the three types of spike pseudoparticles, and their relative infection in percentage was significantly elevated compared to the hESC control (Fig 1O-Q, S1 Fig). Transduction efficiency of the ectoderm and NSCs did not differ significantly from each other for any of the variants. Both ectoderm and NSCs derived from H9 hESCs exhibited elevated susceptibility to SARS-CoV-2 pseudoparticles compared with undifferentiated hESCs.

### TMPRSS2 inhibition in NSCs

Inhibition of TMPRSS2 cleavage activity was evaluated using small molecule inhibitors (Camostat, Nafamostat, Aprotinin, and Ambroxol) (Fig 2A). All inhibitors successfully decreased TMPRSS2 cleavage of the fluorogenic substrate with inhibition ranging from 40–50% (Fig 2A).

**Fig 2:**
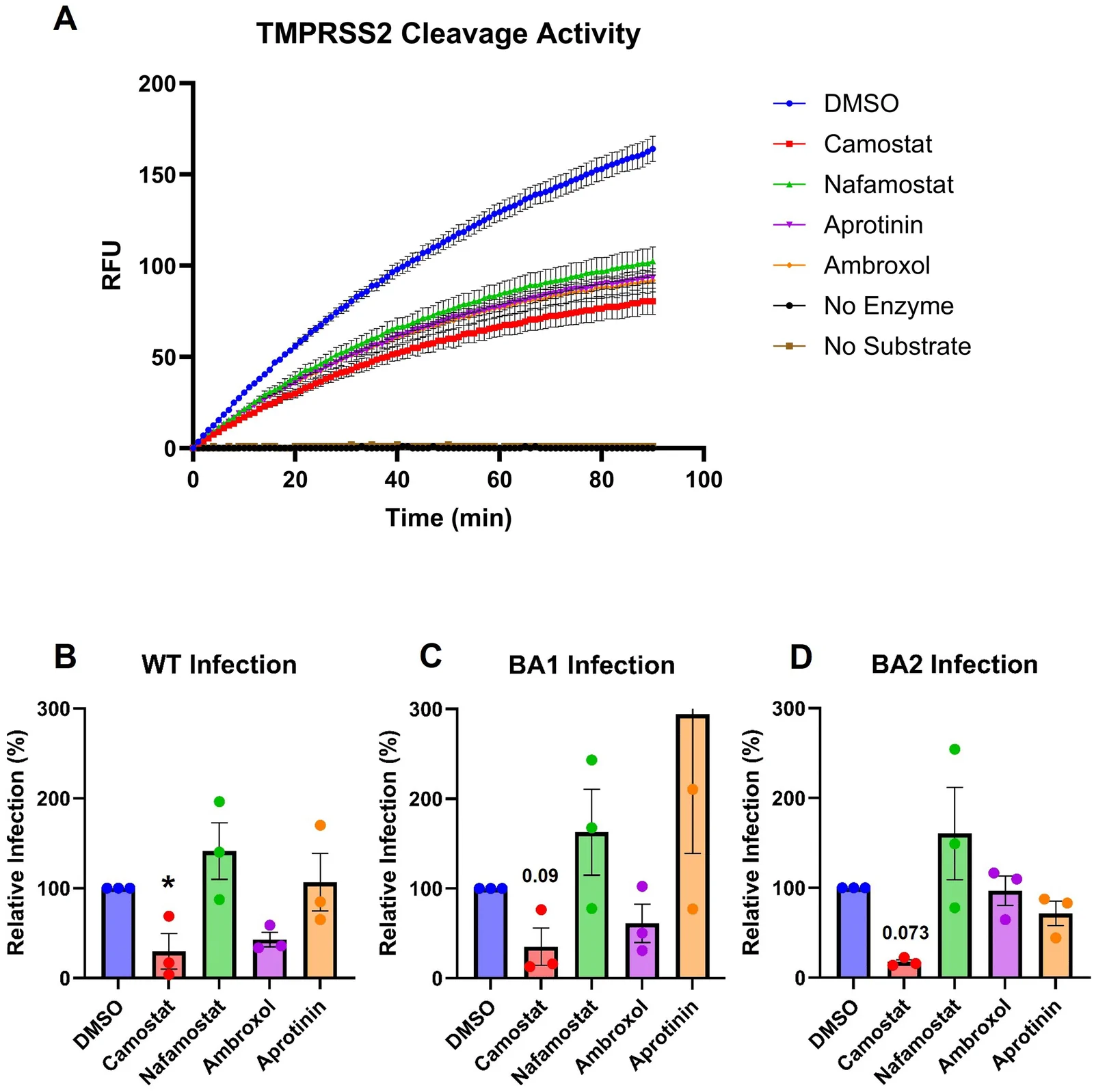
TMPRSS2 inhibitors decreased SARS-CoV-2 entry in NSCs. (A) TMPRSS2 activity of NSCs. RFU = relative fluorescence units. Camostat decreased relative infection with (B) WT Spike pseudoparticles, (C) BA.1 pseudoparticles, and (D) BA.2 pseudoparticles. In (B-D), data were normalized to the DMSO control group. In (C), one data point is above the upper limit of the y-axis (Aprotinin), and the upper limit of the Aprotinin error bar is not shown. Each group is the mean ± SEM of three independent experiments. * = p < 0.05.

Transduction was performed in the presence of TMPRSS2 inhibitors. Relative infection was defined as the transduction efficiency of DMSO control set to 100% (Fig 2B-D). Only camostat decreased infection across WT, BA.1, and BA.2 pseudoparticles. This decrease reached statistical significance for WT (Fig 2B-D) and was close to significant in BA.1 and BA.2. None of the other TMPRSS2 inhibitors significantly affected infection by any of the spike pseudoparticles. The findings with camostat indicate that TMPRSS2 activity contributed to WT spike-mediated entry in NSCs, and probably also contributed to Omicron variant entry.

### Endocytosis-mediated entry and its inhibition

SARS-CoV-2 can also enter cells via endocytosis. Dextran conjugated with TRITC was used to visualize endocytosis in the presence or absence of inhibitors (Dyngo4A, OcTMAB, MiTMAB, Pitstop2, mβCD, nystatin, filipin) (Fig 3A; S2 Fig). The untreated DMSO control successfully endocytosed dextran-TRITC, which appeared as small red puncta inside the cells. NSCs treated with endocytosis inhibitors showed decreased red fluorescence, indicating reduced endocytosis (Fig 3A). NSCs treated with Dyngo4A, OcTMAB, MiTMAB, Pitstop2, nystatin, and filipin showed little fluorescence, whereas mβCD showed some endocytic uptake, indicating less efficient inhibition (Fig 3A).

**Fig 3:**
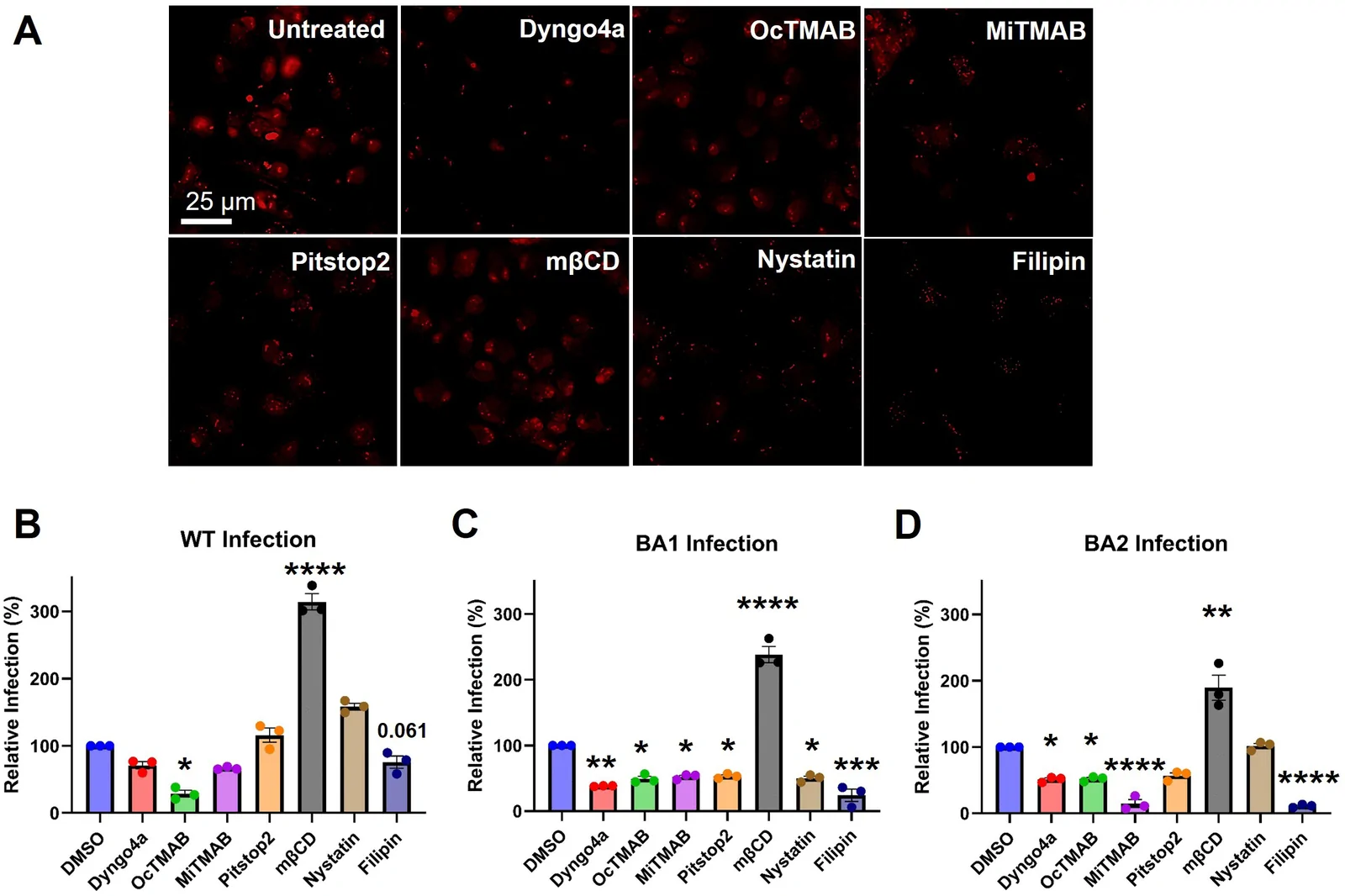
Endocytosis inhibitors decreased SARS-CoV-2 entry in NSCs. (A) All endocytosis inhibitors decreased endocytosis of TRITC conjugated dextran. (B) OcTMAB and Filipin significantly decreased relative infection with WT Spike pseudoparticles. (C) All inhibitors except mβCD significantly decreased relative infection with BA.1 pseudoparticles. (D) Dyngo4a, OcTMAB, MiTMAB, Pitstop2, and Filipin significantly decreased relative infection with BA.2 pseudoparticles. In (B-D), one-way ANOVAs were performed on the raw transduction data, and Dunnett’s post-hoc test was used to compare treated groups to the DMSO control. Data are the means ± SEM of three independent experiments. * = p < 0.05, ** = p < 0.01, *** = p < 0.001, **** = p < 0.0001.

Transduction was next performed with the three types of pseudoparticles and the endocytosis inhibitors (Dyngo4a, OcTMAB, MiTMAB, Pitstop2, Filipin, mβCD, nystatin). Transduction efficiency of the DMSO control was set to 100% and the inhibitor groups were compared to the control (Fig 3B-D). Most endocytosis inhibitors (Dyngo4a, OcTMAB, MiTMAB, Pitstop2, and Filipin) decreased their transduction efficiency relative to the DMSO control, with the exceptions of mβCD and nystatin. For WT spike pseudoparticles, OcTMAB was significantly lower than the DMSO control and filipin was close to significance (*p* = 0.061) (Fig 3B). For BA.1 pseudoparticles, all inhibitors were significantly lower than the DMSO control except mβCD, which was higher (Fig 3C). For BA.2 spike pseudoparticles, all inhibitors were significantly lower than the control except for mβCD and nystatin (Fig 3D). Filipin consistently and significantly reduced infection compared to the DMSO in WT, BA.1, and BA.2 (*p* = 0.061, *p* < 0.001, *p* < 0.0001), in agreement with the fluorescent assay in which filipin was the most effective drug at inhibiting dextran uptake.

Among the tested endocytosis inhibitors, both filipin and OcTMAB significantly decreased transduction efficiency in all three variants. Filipin achieved the highest magnitude of inhibition for BA.1 and BA.2 (75–85% reduction) and WT (50% reduction, p = 0.061). OcTMAB similarly reduced transduction efficiency across variants with statistically significant effects on WT and BA.2 (Fig 3B, D) and the numerically largest effect on BA.1 (Fig 3C). While filipin demonstrated the most dramatic efficacy for Omicron variants, both agents are mechanistically relevant to understanding endocytosis-mediated entry in NSCs. Given this prominent contribution of endocytosis to NSC entry, we next examined whether NSC surface glycocalyx features could modulate receptor access and susceptibility. These data support an important role for dynamin- and cholesterol-dependent endocytic pathways in mediating spike pseudoparticle entry into NSCs.

### Glycocalyx composition and neuraminidase-enhanced entry

To characterize cell surface glycosylation and determine if the glycocalyx affects infectability of the NSCs, glycosylation was assessed using a panel of FITC-conjugated plant lectins with different glycan specificities (Fig 4A-G; S3 A-G Fig). This panel allows spatial and quantitative assessment of predominant glycans on the NSC surface. NSCs exhibited robust binding to Con A, RCA120, and WGA (Fig 4A-C), indicating the presence of abundant terminal mannose, galactose, and sialic acid residues. Limited binding to UEA I, PNA, SBA, and DBA (Fig 4D-G) indicated low surface expression of fucosylated and GalNAc-containing glycans. Based on our previous study [4], we hypothesized that removing glycosylation from the NSCs would increase infectability by providing better access to the ACE2 receptor on the host cell plasma membrane. NSCs were incubated with 0.006 U/mL or 0.018 U/mL of neuraminidase for 0, 45, 90, 180, or 360 min to remove surface sialic acid. Neuraminidase treatment decreased WGA-FITC binding (Fig 4H; S3 H Fig), indicating successful removal of sialic acid groups from the cell surface.

**Fig 4:**
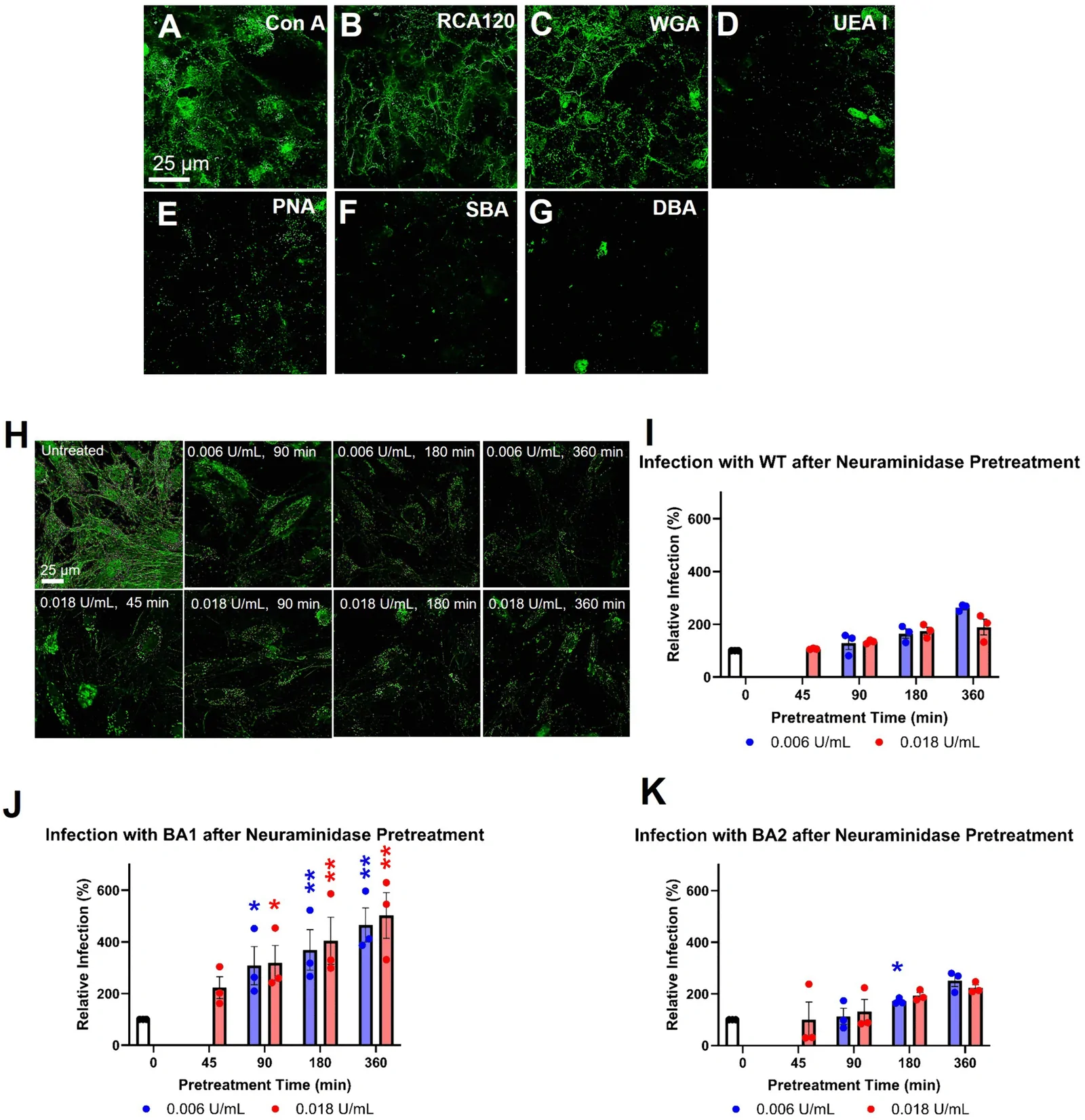
Neuraminidase treatment of NSCs promoted SARS-CoV-2 entry. (A-G) Glycosylation pattern of NSCs was characterized by labeling with FITC-conjugated lectins (ConA, RCA120, WGA, UEA I, PNA, SBA, DBA). Con A recognizes α-linked mannose and glucose; RCA120 recognizes β-galactose; WGA recognizes N-acetyl sialic acid and chitobiose; UEA I recognizes α-linked fucose; PNA recognizes β-galactose in Tn/T antigen contexts; SBA recognizes α- and β-N-acetyl galactosamine; DBA recognizes N-acetyl galactosamine. (H) Neuraminidase treatment (0.006 U/mL, 0.018 U/mL) significantly increased relative infection with: (I) WT, (J) BA.1, and (K) BA.2 pseudoparticles, based on one-way ANOVAs on raw transduction data followed by Dunnett’s post-hoc tests to compare neuraminidase treated groups to the untreated control. Data are the means ± SEM of three independent experiments. * = p < 0.05, ** = p < 0.01, **** = p < 0.0001. Blue asterisk = one-way ANOVA significance for 0.006 U/mL of neuraminidase; Red asterisk = one-way ANOVA significance for 0.018 U/mL of neuraminidase.

To determine if removal of sialic acid would increase SARS-CoV-2 entry, NSCs were pretreated with neuraminidase (0.006 U/mL, 0.018 U/mL) for various periods of time before transduction with SARS-CoV-2 pseudoparticles. For WT and BA.2 spike pseudoparticles, an overall increase in relative infection was observed in the 0.006 U/mL groups, especially, BA.2 pretreated for 180 min was significantly increased; however, in the 360 min groups, relative infection was not significantly different from the control, likely due to some cell death (Fig 4 I K). For BA.1 spike pseudoparticles, relative infection significantly increased as the time of pretreatment with neuraminidase increased, with the higher concentration producing a larger effect faster (Fig 4J). Overall, a positive correlation between the loss of WGA labeling and an increase in relative infection was observed for the three types of pseudoparticles. Fold increases ranged from about 2−4 with BA.2 pseudoparticles having the largest increase. These observations show that both a sparse NSC glycocalyx and enzymatic removal of surface sialic acids from NSCs enhanced SARS-CoV-2 pseudoparticle entry.

## Discussion

Our data provide strong evidence that NSCs, like their ectodermal progenitors [4], are highly susceptible to SARS-CoV-2 pseudoparticles. This vulnerability arises from two principal factors: (1) the ability of NSCs to support both fusion‑ and endocytosis‑based viral entry, and (2) their sparsely developed glycocalyx, which offers limited steric protection against viral attachment. Together, these attributes create a permissive microenvironment for viral invasion and raise concerns that SARS-CoV-2 may perturb the development or function of ectoderm‑derived tissues, including the nervous system and integumentary structures.

The rapid global dominance of Omicron and its subvariants, including the currently prevalent XFG lineage, reflects extensive spike protein mutations that enhance transmissibility and immune evasion [22-24,28–31]. Our results show that NSCs are highly susceptible to both BA.1 and BA.2 Omicron pseudoparticles, raising concerns that these variants may retain or even enhance viral tropism for neuroectodermal tissues. Given Omicron’s virulence and resistance to pre-existing immunity [32,33], heightened vigilance is warranted among pregnant individuals, as *in utero* exposure may pose risks to prenatal neurodevelopment.

Our study investigated viral entry as a potential therapeutic target, consistent with prior proposals emphasizing host-cell entry modulation [34,35]. In NSCs, TMPRSS2 inhibition had limited effects: of the four inhibitors tested, only camostat significantly reduced WT infection, whereas BA.1 and BA.2 infections were not significantly altered despite strong suppression of TMPRSS2 activity (Fig 2B-D). In contrast, endocytosis inhibition produced large, variant-dependent effects: for WT, only OcTMAB significantly decreased infection, whereas for BA.1 and BA.2, most endocytosis inhibitors, including Dyngo4a, OcTMAB, MiTMAB, Pitstop2, and especially filipin, significantly reduced viral entry (Fig 3B-D). Together, these findings support a model in which Omicron variants rely predominantly on endocytosis-mediated entry into NSCs, whereas WT spike can exhibit both TMPRSS2 and endocytosis-based viral entry. Our results are in line with reports that Omicron lineages show reduced TMPRSS2 usage and greater reliance on endosomal entry pathways in other cell types including A549, VeroE6-TMPRSS2, HEC50B, Calu-3, and human nasal epithelial cells [36–39].

OcTMAB and filipin demonstrated their potential as endocytosis entry-targeting antiviral agents. OcTMAB significantly reduced pseudoparticle transduction across all spike variants, consistent with its reported inhibition of dynamin dependent endocytosis [40–42]. Filipin, which disrupts caveolae‑mediated endocytosis by perturbing cholesterol‑rich membrane domains [27,43], similarly blocked SARS-CoV-2 entry particularly for BA.1 and BA.2. In contrast, mβCD did not protect NSCs and instead significantly enhanced their infection, in agreement with prior reports showing it failed to protect against infection using other viruses and other cell lines [44,45]. While our results suggest that OcTMAB and filipin are useful for blocking SARS-CoV-2 entry, further evaluation is required with additional cell types and *in vivo* systems before application to real-word therapeutics.

The cell surface glycocalyx, a dense layer of polysaccharides and glycoproteins, serves multiple functions, including cell recognition, signaling, and mechanical protection, and its integrity has emerged as a critical determinant of SARS-CoV-2 host-cell interactions [46–48]. During viral entry, extensive glycosylation can sterically hinder receptor accessibility and mask the epitopes recognized by viral attachment proteins, whereas cells with sparse glycosylation present more accessible attachment receptors and are therefore generally more susceptible to receptor-mediated viral entry. Our glycosylation profiling demonstrates that NSCs, like their ectodermal progenitors, have a sparse glycocalyx, which increased SARS-CoV-2 pseudoparticle entry relative to non-ectodermal cell types [4]. Consistent with our previous study [4], enzymatic removal of surface glycans using neuraminidase significantly increased relative infection with Omicron variants across most treatment groups, reinforcing the importance of surface glycosylation in modulating viral susceptibility. These host cell determinants likely intersect with variant-specific spike properties, which can bias entry toward TMPRSS2-dependent fusion or endosomal pathways. In endothelial cells, for example, a heparan sulfate–rich glycocalyx can bind spike protein but concurrently shield ACE2, whereas enzymatic shortening or pathological shedding of the glycocalyx exposes ACE2 and enhances spike–ACE2–dependent adhesion and downstream endothelial dysfunction [46–48]. In addition, other groups have shown that glycosylation of the ACE2 host receptor can influence viral entry; for example, glycan shielding on ACE2 may obscure key binding sites, thereby reducing viral entry efficiency [49], and clinical as well as experimental studies indicate that endothelial glycocalyx thinning and shedding of heparan sulfate proteoglycans, such as syndecan-1 and hyaluronan, correlate with COVID-19 severity and microvascular injury [46,47].

Neuraminidase pretreatment significantly enhanced the entry of BA.1 pseudoparticles into NSCs, with a more modest, but significant, effect on BA.2. This difference in BA.1 and BA.2 may be related to the N-terminal domain architecture of BA.1, which contains several deletions and a Glu-Pro-Glu insertion that reshape the exposed N-terminal domain loops and distinguishes it from BA.2 [50]. The N-terminal domain is proposed to interact with sialic acids on the host-cell surface, and several BA.1-specific indels affect residues involved in this interaction [51]. Our results suggest that BA.1 is less able to use terminal sialic acids for productive attachment to the cell surface glycocalyx. In this NSC model, terminal sialic acids act mainly as a physical barrier for BA.1. Their removal by neuraminidase may expose underlying heparan sulfate or ACE2, resulting in a greater increase in BA.1 entry than in BA.2 or WT.

Our data showing that both ectoderm and NSCs exhibited significantly higher pseudoparticle entry than hESCs suggest that derivatives of this lineage may be adversely affected in COVID-19 patients. The medical literature also documents the involvement of the nervous system in COVID-19 [5] with SARS-CoV-2 detected in the brains of deceased patients [52] and in cerebrospinal fluid during autopsies [53]. The expression of ACE2 in the brain, even at low levels, supports the neuroinvasive potential of SARS-CoV-2 [54]. Neurological complications in adults should not be underestimated, as conditions such as Guillain-Barre syndrome [9] and in rare instances, acute encephalopathy [11], have apparently been triggered by SARS-CoV-2 infection. Even asymptomatic adult COVID-19 patients [55,56] report anosmia and ageusia [6,7], suggesting SARS-CoV-2 damage to the olfactory epithelium [57]. Taken together, these findings in conjunction with our data showing elevated pseudoparticle entry into ectoderm and NSCs relative to hESCs suggest that SARS-CoV-2 can target ectodermal derivatives across developmental stages. Accordingly, it is critical that clinicians monitor infants and children born to mothers infected with SARS-CoV-2 during pregnancy for signs of cognitive or neurodevelopmental impairment.

This need for neurological monitoring is underscored by persistent symptoms reported in individuals with post-acute sequelae of SARS-CoV-2 infection (“Long COVID”), including fatigue, cognitive dysfunction (“brain fog”), dyspnea, and sleep disturbances [13,14]. A recent study quantified cognitive impairment in affected individuals, revealing a measurable IQ loss in long-COVID patients experiencing brain fog [58]. In contrast to microcephaly observed in congenital Zika virus infection [59], overt anatomical deficits have not been reported at the time of birth in children born to mothers infected with SARS-CoV-2 during pregnancy [60]. However, a recent large electronic health record cohort confirmed clinical concerns by reporting that maternal SARS‑CoV‑2 infection during pregnancy was associated with increased risks of a neurodevelopmental diagnosis by age three (adjusted OR 1.29, 95% CI 1.05–1.57) [61]. Emerging experimental and clinical evidence support the idea that neuroectodermal development is adversely affected by SARS-CoV-2, which is vertically transmitted from an infected mother to her embryo/fetus.

The present study focused on NSCs differentiated from the H9 hESC line, which was used in our prior work, and which is derived from the human inner cell mass and serves as a widely used, well-characterized reference line for studies of early human development [62–64]. Future studies could determine whether the tropism and entry pathway preferences observed in H9-derived NSCs are conserved when NSCs are derived from iPSC lines, which are not from embryos, or from other hESC lines.

Similar to our prior work with ectoderm, this study demonstrates that NSCs are also more susceptible to SARS-CoV-2 than hESCs, endoderm, and mesoderm. The susceptibility of the ectoderm and NSCs was primarily attributed to the utilization of two viral entry pathways and the sparse glycocalyx on their cell surfaces, which facilitated viral access to the host-cell receptors. The efficacy of entry inhibitors varied with filipin emerging as the most potent agent in NSCs and a potential candidate for further translational investigation. Future studies should monitor the offspring born to SARS-CoV-2–infected mothers for evidence of cognitive and neurological deficits.

## Supporting information

S1 Table List of abbreviations and their definitions. (PDF)

S1 Fig Representative fluorescent images of SARS-CoV-2 pseudoparticle challenged cells. (A) H9 hESCs and (B) NSCs were Zsgreen-positive, indicating successful transduction with WT spike pseudoparticles.
(TIF)

S2 Fig DAPI counterstain of NSCs for Dextran endocytosis assay. Each panel is a DAPI image of the matching Dextran micrographs in Fig 3.
(TIF)

S3 Fig DAPI counterstain of NSCs for glycosylation and deglycosylation assay. Each panel is a DAPI image of the matching glycosylation or deglycosylation assay micrographs in Fig 4.
(TIF)
