# Supplementary material for: Susceptibility of human neural stem cells to SARS-CoV-2: Entry mechanisms and glycocalyx influence
Source: PLoS One. 2026 Oct 21;21(10):e0360734. doi: 10.1371/journal.pone.0360734 (PMC13686471; doi:10.1371/journal.pone.0360734)
Supplement: Supplementary file 1 [file pone.0360734.s001.pdf]

S1 Table. List of abbreviations and their definitions

Abbreviation	Definition
ACE2	Angiotensin-Converting Enzyme 2
ANOVA	Analysis of Variance
COVID-19	Coronavirus Disease 2019
DMEM	Dulbecco's Modified Eagle Medium
DMSO	Dimethyl Sulfoxide
DTT	Dithiothreitol
hESCs	human Embryonic Stem Cells
mβCD	methyl-β-cyclodextrin
MiTMAB	Myristyl trimethyl ammonium bromide
NSCs	Neural Stem Cells
OcTMAB	Octadecyltrimethylammonium Bromide
PBS	Phosphate-Buffered Saline
PFA	Paraformaldehyde
RBD	Receptor Binding Domain
SARS-CoV-2	Severe Acute Respiratory Syndrome Coronavirus 2
TMPRSS2	Transmembrane Protease, Serine 2
